# Development of Texture-Modified Meat and Thickened Soup Combination for Oral Dysphagia Patients with Uniform Firmness and Solid Appearance

**DOI:** 10.3390/foods14142462

**Published:** 2025-07-14

**Authors:** Sergio Hernández, Samuel Verdú, Pau Talens, Raúl Grau

**Affiliations:** Instituto de Ingeniería de Alimentos–FoodUPV, Universitat Politècnica de València, Camino de Vera s/n, 46022 Valencia, Spain; saveram@upvnet.upv.es (S.V.); pautalens@upv.es (P.T.); rgraume@tal.upv.es (R.G.)

**Keywords:** texture-modified foods, dysphagia, IDDSI level 4, transglutaminase, papain, guar gum

## Abstract

This study aimed to improve the visual appeal of texture-modified (TM) dishes for individuals with dysphagia by developing a method to unify the texture of solid and liquid components through innovative food processing techniques. It investigated various meat-softening methods while preserving its solid appearance and ensuring a uniform texture when combined with a thickened soup. A grinding and reconstitution approach enabled the incorporation of pea protein (0% and 1%), olive oil (0%, 5%, or 10%), and papain (0% and 0.2%) to enhance the nutritional and sensory properties. This method successfully matched the firmness of TM meat with that of the thickened soup. Papain significantly reduced the firmness, and olive oil decreased the cohesiveness. After categorizing the TM meat and thickened soup as IDDSI level 4, four dishes at three firmness levels were developed. This study highlighted the potential of this approach to integrate solid and liquid food matrices, contributing to the advancement of TM food engineering and to the challenge of improving visual sensory acceptance and personalizing TM diets for individuals with dysphagia.

## 1. Introduction

Increasing life expectancy and declining birth rate are leading to a rapidly aging population, where the United Nations forecasts that in 2050, 16% of the population will be over 65 years of age [1]. Elderly people can suffer from chewing or swallowing difficulties caused by the deterioration of many functions or the loss of teeth or bone mass [2]. Among them, dysphagia is probably one of the most critical disorders, reaching a prevalence of around 20% in people over 50 years of age and more than 60% in people living in healthcare institutions [3].

Dysphagia can be broadly classified into three major categories: oral, pharyngeal, and esophageal (oral and pharyngeal types are commonly classified together as oropharyngeal in clinical practice) [4]. Oral dysphagia makes it challenging to form safe-to-swallow boluses, which can lead to serious health consequences, such as choking or aspiration pneumonia [4,5]. In response to this, texture-modified (TM) foods are being developed [6]. TM foods are generally prescribed according to the recommendations of speech and language pathologists with experience in oropharyngeal dysphagia, but to obtain a common protocol, the International Diet Dysphagia Standardization Initiative [7] elaborated on a recommendation. They are usually achieved by thickening liquids through the incorporation of proteins and polysaccharides or by grinding solids to obtain purees or creams [8,9,10]. However, these crushed foods have several drawbacks, including unbalanced diets with deficiencies in vitamins, minerals, and protein, which is aggravated by the negative effect from the psychosocial point of view where a diet based on creams or purées decreases appetite [11]. This is a crucial aspect since elderly people with swallowing and chewing difficulties often suffer from malnutrition, which is characterized by deficiencies in energy and protein intake, among other nutrients. This is mainly due to a decreased appetite and physiological changes that make it difficult to consume many protein-rich foods [12,13]. Miles et al. [14] reported that the energy intake of people on TM diets tends to be 17–37% lower than that of usual diets.

Innovative texture-modifying techniques are emerging to improve visually appetizing food [15]. Three-dimensional food printing and the enzyme-softening treatment are examples of this, as they enable smooth textures while maintaining an appearance similar to that of ordinary foods [16,17,18]. Three-dimensional printing is especially popular because it can make food look more appealing. However, it has some small drawbacks in terms of efficiency, printing accuracy, and keeping nutritional qualities consistent [13]. The enzyme-softening treatment has been widely applied due to its capacity to preserve the original shape, resulting in products that are very tender [19]. In addition, grinding followed by enzymatic reconstitution is beginning to be investigated due to its potential to produce texture-modified (TM) foods that are very soft, homogeneous, and have a solid appearance. Moreover, this technique allows for the addition of substances that improve the product both nutritionally and technologically, enabling the customization of the nutritional content of the dishes [20].

Along this line, the design of dishes, including different food matrices with a solid appearance, could improve the appetizing and nutritional profile of the dishes. The main challenge of this strategy is to homogenize the texture and reduce the risk of choking when a bolus forms that can be swallowed [21]. However, this strategy has already been used to incorporate a texture-modified vegetable, treated with enzymes, into a thickened soup [21]. Nevertheless, its application to meat products has not yet been explored. In this sense, once all this has been achieved, developing dishes with different levels of texture and nutritional intakes to personalize these TM dishes could be an essential step forward.

Therefore, the main objective of this study was to deepen the understanding of strategies for combining different matrices with homogeneous textures from an engineering perspective. The central hypothesis was that it is possible to obtain solid and liquid components with similar firmnesses, and that this strategy could be applied to improve the visual appeal of dishes designed for individuals with dysphagia. To address this, the secondary objectives were (1) to explore methods for producing the softest possible meat texture while preserving its solid appearance; (2) to develop a thickened soup (TS) with a firmness comparable with that of the texture-modified (TM) meat; (3) to analyze the effects of thickeners on the final product; and (4) to evaluate the textural properties of dishes that combined both the TM meat and TS.

## 2. Materials and Methods

### 2.1. Materials and Reagents

Fresh pork loin (*Longissimus dorsi*) and commercial vegetable and chicken soup (fat < 0.5 g, saturated fats < 0.1 g; carbohydrates 1.3 g; sugars < 0.5 g; dietary fiber < 0.5 g; proteins < 0.5 g; salt 0.71 g) were purchased from a local Spanish supermarket. Guar gum (GG) (EPSA, Valencia, Spain) was selected based on previous studies because of its thickening properties [21]. Food grade papain (30,000 USP activity), used as a proteolytic enzyme [17], and transglutaminase (TGase) (Probind^®^ MB 1.0–50 TGU/g) were purchased from Biocon S.L. (Les Franqueses de Vallés, Spain). Pea protein (80% protein) was purchased from Laboratorios Almond S.L. (Murcia, Spain).

### 2.2. Experimental Procedure

**Figure 1:**
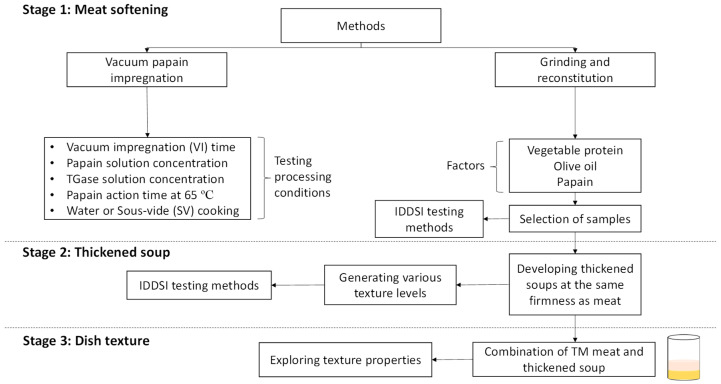
Scheme of experimental procedure.

The study comprised three stages (Figure 1). The first stage consisted of obtaining TM meat samples with a firmness that could be reached by a TS while maintaining a solid appearance. Two treatments were tested: (1) vacuum papain impregnation and (2) grinding and reconstitution using papain and TGase. The treatment with the best results in terms of their texture properties, while maintaining a solid visual appearance, was selected for the next stage. In the second stage, the soup was thickened to the same firmness as the selected TM meat. For this purpose, the guar gum concentration was evaluated. Once both the TM meat and TS were obtained, they were analyzed according to the IDDSI methods. In the third stage, dishes were developed as a combination of both components (TM meat over TS) at different texture levels, and a texture analysis was carried out.

#### 2.2.1. Meat Treatment

##### Vacuum Papain Impregnation

The meat was softened by vacuum enzyme impregnation following the process described by Grau et al. [17] with slight modifications. The samples were frozen at −20 °C for a minimum of 48 h and thawed to enhance the impregnation process. Then, the samples were cut into squares of 2 cm on each side and 1 cm in thickness and were impregnated with papain solution under vacuum. The vacuum impregnation system was designed using a desiccator hermetic chamber equipped with a pump and a manometer. Different processing conditions were tested: (i) vacuum impregnation (VI) time (5 or 30 min); (ii) papain solution concentration (1 or 5% *w*/*v*); (iii) TGase solution concentration (0 or 2% *w*/*v*); and (iv) papain action time at 65 °C (5 min–30 min) with (v) water (W) or sous-vide (SV) cooking. This was followed by boiling at 95 °C for 3 min to inactivate the enzymes. Both the enzyme reaction and inactivation were carried out in a thermostatic water bath (Fisher Scientific, Brussels, Belgium). For the sous-vide cooking, the samples were vacuum-sealed using an industrial vacuum-packaging machine (Tecnotrip, Barcelona, Spain). Six replicates per sample were analyzed.

##### Grinding and Enzyme Reconstitution

The second method employed to soften the meat was by grinding and reconstituting using TGase to give it a solid appearance. Taking advantage of the reconstitution, other ingredients, such as vegetable pea protein, olive oil, and papain enzyme, were added to the meat to improve both its nutritional and organoleptic quality. Pea protein (80% protein) was added because of its high-quality protein [22]. Olive oil was included to address the hypocaloric status and contribute to texture softening, as well as for its bioactive compounds and well-established cardioprotective effects [23,24]. Papain was used to soften the meat’s texture, and TGase was used to reconstitute a solid appearance following previous studies in which both TGase and papain were used at the same time [20].

First, 200 g of pork meat was cut into small pieces and ground for 3 min in a meat grinder (Philips, Eindhoven, The Netherlands). Then, the ingredients were added and mixed in the proportions described in Table 1. Finally, the mixes were reconstituted into cylinders 35 mm in diameter and 10 mm thick. After elaborating, the samples were stored for 24 h at 4 °C for the TGase to act [25]. Then, the samples were placed in an oven at 80 °C for 20 min. The cooking conditions were enough to initially accelerate the papain enzyme activity until the meat was partially tenderized [17,26]. After cooking, the samples were cooled in an ice bath. The overall process and the components, as well as their concentrations, were similar to those used by Ribeiro et al. [20]. All measurements were performed in duplicate.

#### 2.2.2. Thickened Soups

TSs were prepared following the method reported by Hernández et al. [21]. GG at different concentrations was added to the soup at 70 °C, and the solution was stirred for 45 s. First, a calibration curve was prepared using GG concentrations between 2 and 10%. Once the TS firmness model was developed, the concentrations of GG required to achieve the firmness of the different meat samples obtained in the results (6.43%, 7.05%, and 7.9%) were selected. All measurements were performed in duplicate.

### 2.3. Texture Analysis

The texture of the samples was characterized by a back extrusion assay following the conditions used by Hernández et al. [21], with slight modifications. This test was selected because it is a recommended test for semi-solid foods [27]. Measurements were taken by a TA-TX2 texture analyzer (Stable Micro Systems, Surrey, UK) equipped with a 25 kg load cell. The samples were extruded by a cylindrical back extrusion disc (20 mm in diameter) until 80% deformation at a test probe speed of 1.00 mm/s in cells 35 mm in diameter. The TM meat and TS samples had a thickness of 10 mm, while the combination of both was 20 mm, with the TS located below and the TM meat above. The arrangement of the samples was established with the objective of simulating the expected consumption since the TM meat is intended to be arranged over the TS in a dish. The parameters analyzed were the positive peak force (N), negative peak force (N), positive area (N/m^2^), and negative area (N/m^2^), which are correlated with the firmness, cohesiveness, consistency, and viscosity index, respectively [28].

### 2.4. IDDSI Testing Methods

This study conducted tests based on the guidelines provided by the International Diet Dysphagia Standardization Initiative (2019) to establish standardized texture and consistencies for dysphagia diets across eight continuous levels (0–7) in accordance with individual patient restrictions. Levels 0–4 represent drinks, and 3–7 represent foods [25]. The objective was to assess the safety of samples and categorize them into their respective IDDSI levels. As the IDDSI level increases, the difficulty of consumption also increases, requiring greater swallowing ability to consume it without posing a risk to the patient [29]. The testing methods for levels 4 and 5 (fork drip test, spoon tilt test, and fork pressure test) [18,30] were carried out on selected TM meat and TS samples.

### 2.5. Statistical Analysis

At stage 1, the grinding and reconstitution variable firmness was studied by a multifactorial ANOVA (pea protein, olive oil, papain concentrations). In addition, these samples were compared according to all the instrumental texture parameters by employing the multivariate statistical method Principal Component Analysis (PCA).

At stage 2, the factors type of sample (TM meat, TS, and dish) and texture group (1–3) were studied by a multifactorial ANOVA for each texture parameter.

For both stages, the results are reported as mean values with standard deviations. In those cases in which the effect was significant (*p*-value < 0.05), the means were compared by Fisher’s least significant difference (LSD) procedure. The programs employed were Statgraphics Centurion XVII.II, version 17.2.04, and the PLS Toolbox, 6.3 (Eigenvector Research Inc., Wenatchee, Washington, DC, USA), a toolbox extension in the Matlab 7.6 computational environment (The Mathworks, Natick, MA, USA).

## 3. Results and Discussion

### 3.1. Stage 1: Meat Softening

#### 3.1.1. Vacuum Papain Impregnation

**Figure 2:**
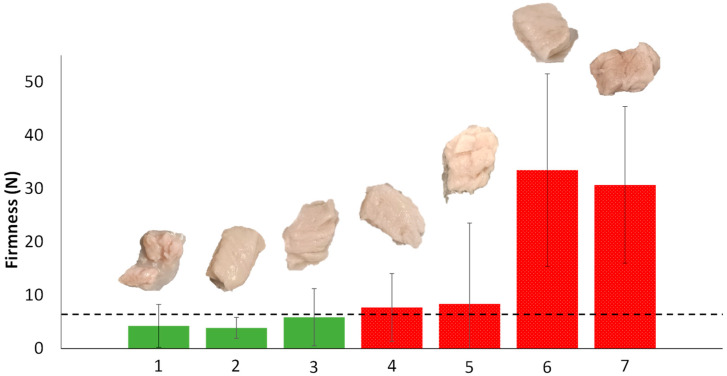
Firmness values of representative vacuum-papain-impregnated meat samples with standard deviations (n = 6) and corresponding images. Green bars indicate samples with adequate firmness, and red bars with dot filling indicate samples with excessive firmness. Processing conditions of samples: 1—VI time: 30 min, TGase: 0%, papain: 5%, papain action time: 10 min, W; 2—VI time: 30 min, TGase: 0%, papain: 5%, papain action time: 15 min, W; 3—VI time: 5 min, TGase: 0%, papain: 5%, papain action time: 10 min, SV; 4—VI time: 5 min, TGase: 0%, papain: 5%, papain action time: 15 min, SV; 5—VI time: 5 min, TGase: 0%, papain: 1%, papain action time: 15 min, W; 6—VI time: 5 min, TGase: 2%, papain: 1%, papain action time: 10 min, SV; 7—VI time: 5 min, TGase: 2%, papain: 1%, papain action time: 10 min, W.

Representative sample firmness values are given in Figure 2. Although this technique has previously been used to soften meat matrices [17,31], it is likely to have some limitations in tenderizing meat to the texture levels attainable by a TS classified as level 4, pureed foods, by the International Diet Dysphagia Standardization Initiative [7] classifying method. According to previous studies by Hernández et al. [21], the firmness limit was established at 6 N (dotted dashed line of Figure 2). These values are close to those observed for tongue strength by other authors [32,33]. Thus, the samples that maintained a solid appearance did not achieve the target firmness levels, where they exhibited excessive hardness (samples of Figure 2 with red bars). Conversely, the remaining samples reached the desired textural parameters but failed to maintain structural integrity since they suffered fraying of their fibers and elongation of their connective tissues (samples of Figure 2 with green bars). Moreover, the results had a high dispersion due to the fraying effect of the samples, which introduced much variability in the measurements. This variability increases the risk of choking when a swallowable bolus is formed. Due to these results, this softening technique was discarded before the next stage.

#### 3.1.2. Grinding and Reconstitution

**Figure 3:**
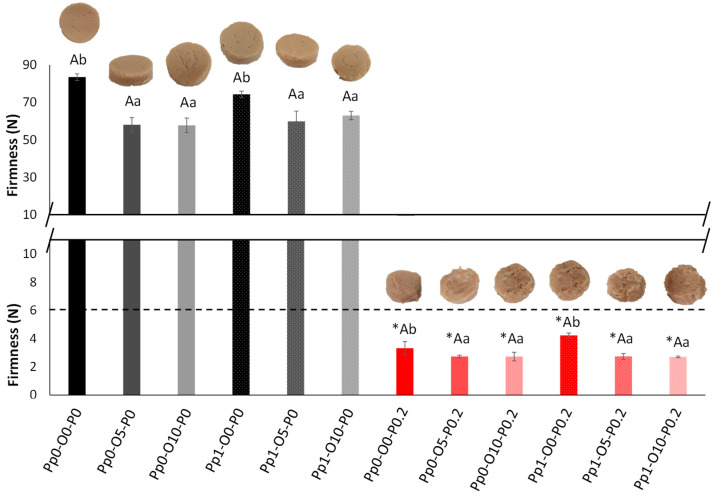
Firmness values of the ground and reconstituted meat samples with standard deviations (n = 2). Black bars corresponded to samples with 0% papain and red bars to samples with 0.2% papain. Bars with a dark color (black or red) indicate samples with 0% olive oil, bars with medium color indicate samples with 5% olive oil, and pale bars indicate samples with 0% olive oil. The white dot filling in bars indicates samples with 1% of protein added. *: significant difference because of papain. Capital letters: significant differences because of protein content. Lowercase letters: significant differences because of olive oil content.

First, the effect of the factors (pea protein, olive oil, papain concentrations), as well as their interactions on the firmness of the ground and reconstituted meat was studied by a multifactorial ANOVA (F-ratios and p-values are included in Table S1 in the Supplementary Information), and the firmness values are plotted in Figure 3.

The concentration of pea protein had a non-significant effect on the firmness of the samples, whereas the concentrations of olive oil and papain had significant effects. The effect of papain was the most significant, with an F-ratio of 2002.49, significantly higher than the olive oil effect F-ratio of 23.03. The presence of olive oil decreased the meat’s firmness. However, no significant differences were observed between the 5% and 10% concentrations, suggesting that the softening effect plateaued after 5%. These results fall in line with those reported by Lu et al. [34] when investigating the effect of incorporating olive oil on the physicochemical properties of surimi gels. On the other hand, the papain addition produced a sharp reduction in the firmness values of the samples (red bars of Figure 3). This was due to the degradation of myofibrillar and connective tissue proteins by papain, which breaks the peptide bonds of lysine, phenylalanine, and arginine [35]. This enzymatic hydrolysis could lead to a possible release of bioactive compounds or to an increase in protein digestibility due to the papain effect of hydrolyzing peptides into peptides with low-molecular-weight sequences [36]. This fact is important in elderly consumption because of their limited gastrointestinal functions, which causes macronutrient maldigestion and malabsorption [37]. As expected, the papain-treated samples with olive oil were the softest samples, but in all cases, the solid appearance was maintained. Nevertheless, a significant interaction (*p*-value = 0.0002) between olive oil and papain was observed. Olive oil reduced the firmness more noticeably in samples without papain, while its effect was less evident when the papain was present. This suggests that papain’s strong proteolytic action dominated the texture degradation and possibly reached a point where additional softening by oil was minimal. Within the samples treated with papain, the samples with the highest firmness were the samples without olive oil and pea protein added (Pp1-A0-P0.2), although these differences were not statistically significant. This fact suggests that in the TM meat samples without olive oil added, the protein incorporation could increase the number of molecular associations [38] since this difference in firmness was not observed between the samples with olive oil added. Although the combined use of papain and TGase has not been extensively studied, the results indicate that this enzymatic combination leads to a marked reduction in firmness while preserving the structural integrity of restructured meat samples. TGase likely contributed to the formation of a cohesive protein matrix, which promoted a solid appearance and enhances water retention, as previously described by [20]. This effect is evident in the samples treated with TGase alone, which exhibited excessive firmness. However, when combined with papain, the higher water content retained within the matrix appeared to counteract this firmness, which resulted in a softer texture due to the joint effects of proteolysis and increased water-holding capacity, while still maintaining a cohesive, structured appearance. This synergistic interaction, first reported by [20], suggests a promising strategy for producing soft yet well-structured TM meat products.

**Figure 4:**
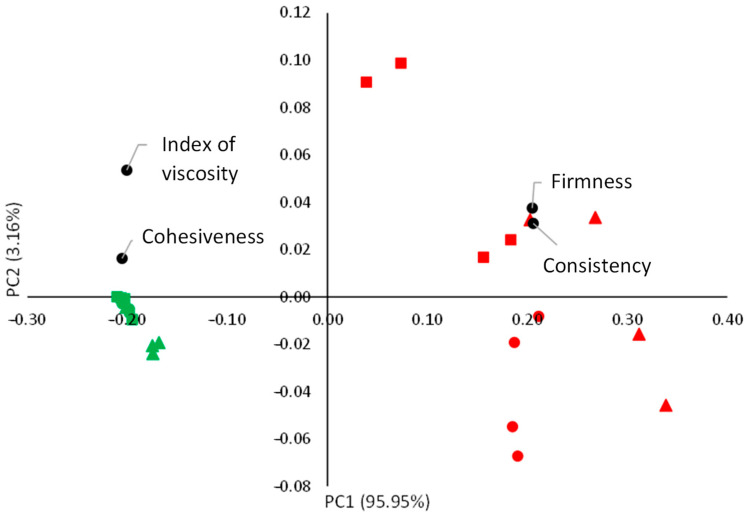
Biplot of PC1 (95.95%) and PC2 (3.16%) from the PCA performed on the texture data for the ground and reconstituted meat samples. The plot displays the average sample scores, as well as the loadings of the main back extrusion parameters. Samples with 0.2% papain (red symbols); samples without papain (green symbols); samples without olive oil (triangle symbols); samples with 5% olive oil (circle symbols); samples with 10% olive oil (square symbols); back extrusion parameters (black symbols).

Multivariate PCA was carried out to explore more parameters of the back extrusion assay and try to characterize the samples more deeply before selecting between these samples for the next step (Figure 4). The PCA reduced the four textural parameters analyzed (firmness, cohesiveness, consistency, and viscosity index) to two variables (linear combinations of the original ones) called PC1 and PC2, which collected 95.9% and 3.2% of the total variance, respectively. The data matrix used for the PCA is included in Table S3 in the Supplementary Information. Figure 4 shows the biplot of the space of variance generated between PC1 and PC2, where the averages of scores are represented, as well as the resulting loadings.

The PCA showed sharply defined clusters because the papain incorporation produced large changes in the firmness, as discussed above. This can be seen in PC1, whereas PC2 could collect the variance fraction provided by the rest of the back extrusion parameters. In this way, PC2 showed the effect of the added olive oil. The samples with olive oil added had higher values of PC2. This was probably because the samples without olive oil had lower values in the negative peak force and negative area and, therefore, had higher cohesiveness [39].

To select the samples for the next stage, the samples with a firmness higher than 6 N were discarded to ensure safe conditions for the elderly with swallowing or chewing difficulties, as explained above. In addition, the samples with 10% olive oil were discarded to pass to the next phase to reduce consumers’ fat consumption on the basis that these samples showed no texture difference according to the ANOVA and PCA relative to the samples with 5% olive oil. Thus, the Pp0-O5-P0.2, Pp1-O5-P0.2 (renamed as groups 1A and 1B, respectively), Pp0-O0-P0.2 (group 2), and Pp1-O0-P0.2 (group 3) samples with Fmax = 2.74, 2.74, 3.32, and 4.23 N, respectively, were selected for the next stage. In this sense, analyzing samples with different texture levels and compositions is an interesting aspect since not all elderly with swallowing or chewing deficiencies have the same restrictions and requirements. The samples with pea protein could be beneficial for populations with protein and specific amino acid deficiencies but not necessary for elderly consumers with suitable protein intake. The same thing is true for olive oil, the consumption of which could be advantageous for people with a low caloric intake, as commented above.

### 3.2. Stage 2: Thickened Soup

**Figure 5:**
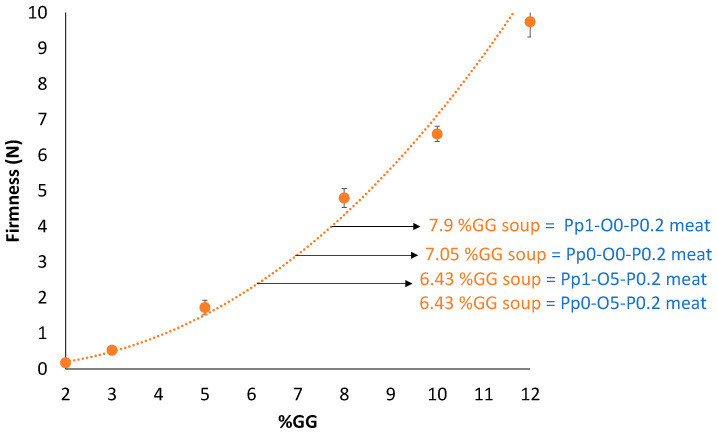
Evolution of TS firmness as function of GG concentration (n = 2).

Once the TM meat samples with adequate firmness were developed, the objective was to equalize the firmness of the TS to these TM meat samples. First, to estimate the GG concentration required to achieve the suitable maximum force for the soup, a calibration curve dependent on the GG concentration was carried out (Figure 5), and a predictive equation (Equation (1)) with a regression coefficient of 0.992 for the maximum force values of the TS was developed:

(1) $$y={0.0416x}^{2.2337}$$

where *y* is the firmness and *x* is the GG concentration.

Once the predictive equation was developed, the required GG concentration for each soup to equalize the firmness with each TM meat sample was calculated and added. For groups 1A and 1B, the GG concentration was 6.43% GG in the soup, 7.05% for group 2, and 7.9% for group 3.

### 3.3. Texture Properties Comparison of TM Meat and TS

**Figure 6:**
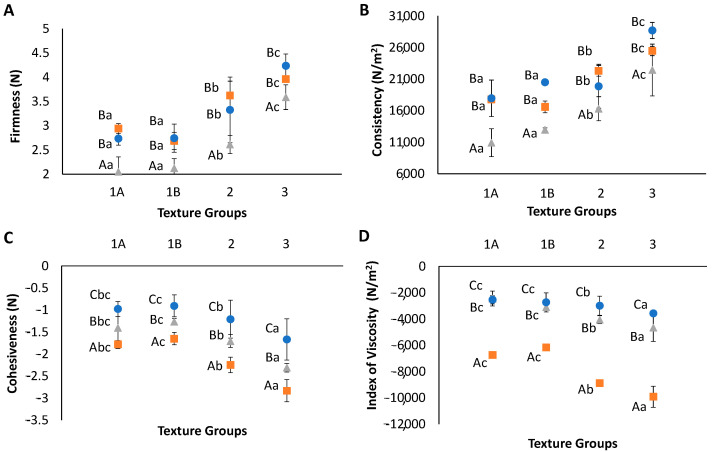
Evolution of the firmness (**A**), consistency (**B**), cohesiveness (**C**), and viscosity index (**D**) for the TM meat samples (blue points), TS (orange points), and combination of both (grey points) with standard deviations (n = 3) as a function of the texture group. Capital letters indicate significant differences within each texture group as a function of the sample type. Lowercase letters indicate differences within each sample type as a function of the texture group.

After equalizing the firmness of both components, the back extrusion parameters of both components were compared to delve into their textural properties. These results are plotted in Figure 6 (F-ratios and p-values are included in Table S2 in the Supplementary Information). As expected, the TS firmness values were consistent with the theoretical calculations performed previously, and no statistical differences were demonstrated between the firmnesses of the TM meat and TS in all three developed textures. No statistically significant differences in the firmnesses were observed between texture groups 1A and 1B, whereas it increased significantly in texture groups 2 and 3 (Figure 6A). The same behavior was observed for consistency (Figure 6B). Nevertheless, significant differences were observed between both components (TM meat and TS) for cohesiveness and viscosity index in the three textures (Figure 6C,D). The cohesiveness and viscosity index had similar performances. In the case of the TS, both parameters increased significantly for texture groups 2 and 3, while in the case of the TM meat, the meat cohesiveness also increased significantly, although to a lesser extent than in the TS, especially for the viscosity index. The TM meat cohesiveness increased in texture groups 2 and 3, probably due to the absence of olive oil, and in the case of texture group 3, this was in addition to the effect of incorporating the pea protein explained above.

The differences observed for the cohesiveness and index of viscosity between the TM meat and TS in the three textures were probably because although the firmness and consistency of both components were equalized, these components were not the same product, and they had many structural differences. The TS had a significantly higher cohesiveness and viscosity index than the TM meat. Cohesiveness indicates the force required to overcome the internal force of the food-attracting molecules and is also related to adhesiveness because it coincides with the force required to separate the compression disc and the sample [40]. The viscosity index is expressed as the “work of cohesion”. This means that in spite of the TM meat and TS having similar firmnesses and consistencies, the TS generated more resistance to the withdrawal of the sample from the disc due to their higher cohesion between internal molecules and viscosity [39]. This was one of the main limitations of the study, which is explored in the remainder of the manuscript.

### 3.4. IDDSI Methods

**Figure 7:**
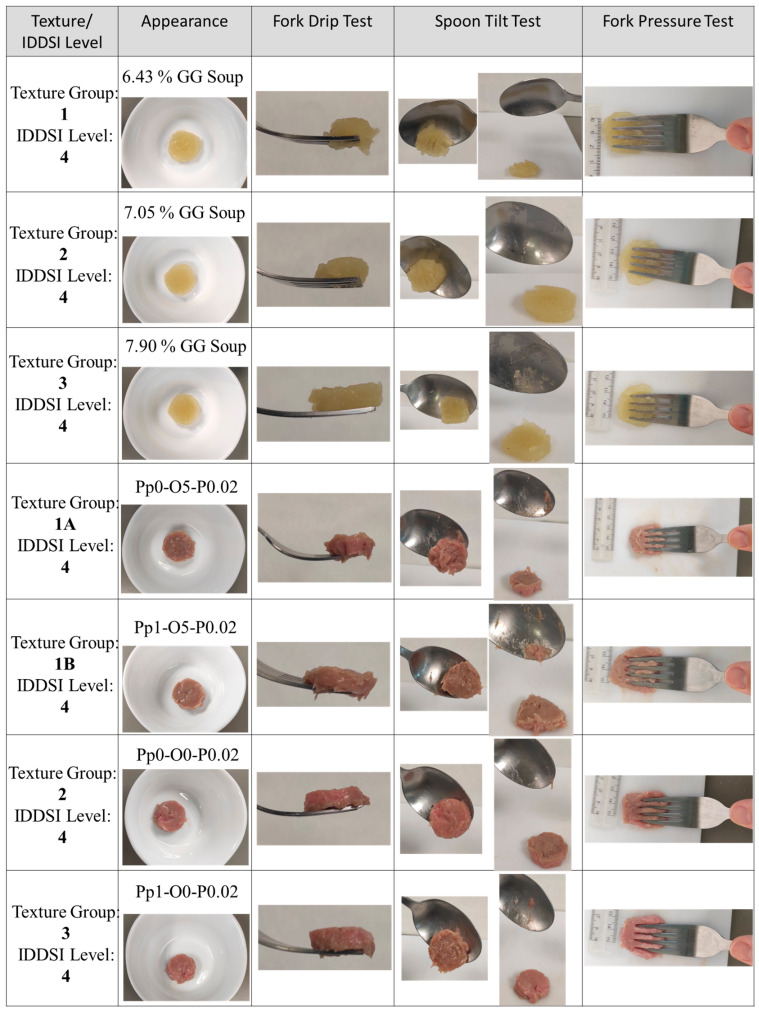
IDDSI tests conducted on selected TM meat samples and TS.

Once the texture for both compounds had been evaluated, the next step was to evaluate their classification according to IDDSI testing methods. Images of the IDDSI testing methods conducted on the selected TM meat and TS samples are collected in Figure 7. The fork drip test was used to evaluate their consistency by seeing whether the sample passed the prongs of the fork [29]. The samples exhibited adequate consistency as they did not flow to the prongs and were maintained together on the fork.

The spoon tilt test was employed to determine their stickiness and cohesiveness by tilting the spoon until the samples fell and interpreting their behavior [41]. Figure 7 shows that the samples were able to maintain their shape on the spoon and slide out of the spoon with little food remaining by turning the spoon laterally [42].

The fork pressure test was performed by applying force with a fork using the thumb and observing the textural behavior and changes in the samples. During the application of fork pressure, the samples did not cause any blanching on the thumbnail, and the particles were easily separated by the fork’s prongs. This is an important aspect since if the particles have difficulties passing through the fork’s prongs (4 mm), these samples could imply a risk of asphyxiation. In addition, this test confirmed the lack of lumps, and a clear pattern could be appreciated. For this reason and for the rest of the IDDSI test results discussed below, the samples were classified as IDDSI level 4 (pureed and extremely thick products), similar to Dhillon et al. [29] and Zhu et al. [43].

### 3.5. Stage 3: Dish Texture

The results of both components measured together (dish) by placing the TS under the TM meat are shown in Figure 6 (grey triangle symbols). The dish firmness decreased significantly compared with the individual plate component measurements in the three texture groups developed (Figure 6A). The decrease could have been due to the interaction that occurred between the TM meat and the TS, as has already been reported by Hernández et al. [21]. The component located above could transfer force to the component below, which could absorb the force and begin to deteriorate as the cylindrical back extrusion disc came into contact with the component above. As expected, the dish firmness increased significantly in texture groups 2 and 3. The consistency showed similar behavior to the firmness, which resulted in the dish’s consistencies being significantly lower than the TM meat and TS consistencies and being higher in texture groups 2 and 3 than in group 1 (Figure 6B). The consistency of the dish with texture group 3 was significantly higher than the consistency of the dish with texture group 2. The dish’s cohesiveness resulted in a mixture of both components having a cohesiveness significantly higher than the TM meat and significantly lower than the TS (Figure 6C). Like the individual measurements, the cohesiveness of the dish samples increased significantly in texture groups 2 and 3. Similar behavior to the cohesiveness was observed for the viscosity index, with the difference that the dish viscosity index values were more like the TM meat than the TS, so the TM meat had more loading in the viscosity index than the TS. Thus, like the TS, the viscosity index increased significantly for texture groups 2 and 3.

**Figure 8:**
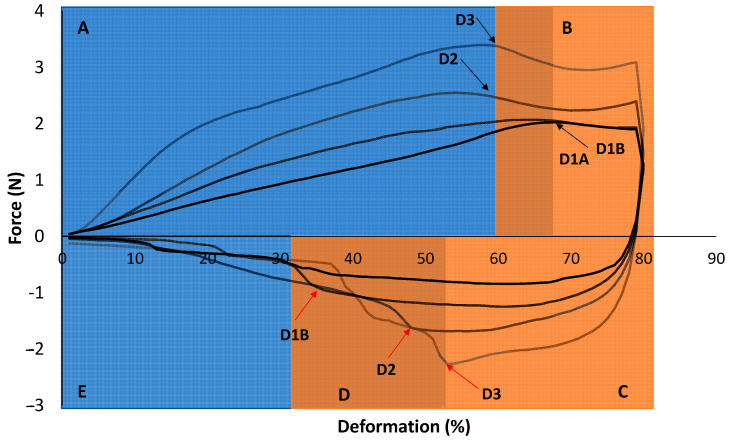
Back extrusion average curves (n = 3) for dishes with texture group 1A (D1A—black line), dishes with texture group 1B (D1B—dark grey line), dishes with texture group 2 (D2—grey line), and dishes with texture group 3 (D3—pale grey line). Black arrows indicate the point where the disc encountered the TS during the descent. Red arrows indicate the point where the disc encountered the TM meat during the upward flow. The letters A–E indicate each of the five phases into which the back extrusion test was divided.

To understand the different behavior of the complete dish in comparison with its individual components and to gain insight into its potential performance during oral processing, the average back extrusion curves were analyzed (Figure 8).

The exploration of the profile of the back extrusion curves was divided into five phases based on the matrices that were traversed by the disc at each time point. Phase A corresponded with the zone where the disc passed through the TM meat. This phase had a typical non-linear region with a slight increase in the slope produced at the beginning of the measurement, as characterized by several authors [27]. This increase in the slope was more pronounced for the dishes with texture groups 2 and 3 than for the dishes with texture group 1 since the greater the increase in the slope, the greater the stiffness/elasticity ratio [44]. Then, the disc continued extruding the TM meat, and a linear region was observed until it arrived at the convergence of both matrices (phase B). This convergence point is marked in Figure 8 with a black arrow joined to the respective name of each dish. The dishes with texture groups 1A and 1B showed a slower convergence due to their higher elasticity and lower stiffness relative to the dishes with texture groups 2 and 3. This point produced a slight reduction in the registered force, which was almost non-perceptible for the texture groups 1A and 1B dishes. The disc continued going down to 80% deformation, where the curves of the dishes with texture groups 2 and 3 registered a distinguishable peak force, which has also been characterized by some authors [27]. However, the dishes with texture groups 1A and 1B did not display this peak force because of their high homogeneity and lower firmness. Next, the disc started to return, and the cohesiveness and viscosity index parameters started to affect the curve (phase C). This phase corresponded with large negative force values produced by the cohesiveness of TS; these values were significantly higher for the dishes with texture groups 2 and 3, as commented above (Section 3.3). The next phase (phase D) demonstrated the most changes in the slope of the curves due to this zone corresponding with the zone where the negative parts of both matrices converged, and as has been previously explained, these components had differences in their cohesiveness and viscosity index. The red arrows in Figure 8 highlight the region of the curve where the disc came in contact with the TM meat, which generated a marked change in the slope for the dishes with texture groups 2 and 3 and showed slight differences between both dishes with texture groups 1A and 1B. While for dish Pp1-O5-P0.2 (texture group 1B), a subtle change was observed, a non-perceptible change was observed for Pp0-O5-P0.2 (texture group 1A). Finally, when the disc passed 35% of the deformation (phase E), only the TM meat negative force was registered, which generated a horizontal slope.

This exploration of the texture measurement curves allowed us to confirm that the dishes categorized as texture groups 1A and 1B exhibited more homogeneous textural profiles compared with those in groups 2 and 3. Such a consistency is particularly relevant for individuals with moderate-to-severe dysphagia, as it suggests that these formulations may be safer and more acceptable for consumption by patients with more restrictive swallowing capacities.

Moreover, the relatively uniform behavior of components within texture groups 1A and 1B, as reflected by the nearly horizontal slope of the back extrusion curves, even in the later compression phase (phase D), suggests that these dishes underwent minimal resistance changes during oral processing. This stability may translate into a smoother and more predictable sensory experience, potentially reducing fatigue and anxiety during eating in dysphagic patients [45].

Interestingly, the results also suggest that the simultaneous consumption of multiple components within a single dish could contribute to a compensatory effect, in which differences in the cohesiveness and viscosity index are partially balanced. This textural smoothing effect is especially evident in dishes with more uniform matrices (1A and 1B), and may help improve bolus cohesion and transport, which are both critical factors for safe swallowing [46].

Taken together, these findings underscore the potential of texture-modified composite dishes as tailored nutritional solutions for people with dysphagia. They highlight the importance of not only optimizing individual components but also considering the interactive effects when consumed together, which may further enhance the safety, palatability, and nutritional compliance in clinical populations.

A limitation of the present study was the absence of rheological profiling and the lack of sensory evaluation. While the texture optimization was performed using back extrusion to ensure safety, consistency, and structural compatibility with thickened soups, further texture studies with more realistic approximations of human masticatory processes, alongside rheograms and organoleptic assessments, are required. These efforts will improve the clinical relevance, user acceptability, and practical design and development of texture-modified (TM) foods for elderly populations with specific chewing and swallowing disorders.

## 4. Conclusions

Throughout the evaluation of two different meat-softening methods, (i) vacuum papain impregnation and (ii) grinding and reconstituting meat with papain and TGase, only the latter was found to be effective at obtaining softened meat to IDDSI level 4 while maintaining structural integrity to provide a solid appearance. This method allows for the personalization of dishes by adding ingredients such as pea protein and olive oil to improve the nutritional and organoleptic properties of the dishes. The addition of papain significantly reduced the firmness of the meat, while olive oil decreased the cohesiveness, which contributed to the desired texture. Four texture-homogeneous dishes were developed, which consisted of texture-modified meat and a thickened soup that maintained a solid appearance. These dishes were formulated at three distinct firmness levels and with varying nutritional profiles, which offered a step toward more tailored dysphagia-friendly products, although further sensory validation is needed. Although the dishes were prepared to have the same firmness, the measurement of both components independently showed differences in the cohesiveness and viscosity index; nevertheless, when they were measured when combined, the differences were smoothed out, especially for the texture groups 1A and 1B dishes. Further studies are needed to delve deeper into oral processing in healthy and unhealthy consumers in order to develop high-quality and safe swallowable texture-modified foods in a more personalized way. Additionally, it is essential to evaluate consumer acceptance through sensory analysis to ensure that these formulations meet both functional and sensory expectations. Furthermore, additional research is needed to evaluate and optimize the potential industrial application of this approach, including microbiological validation, for practical implementation in the food industry.

## Figures and Tables

**Table 1:** Experimental design and formulations (Pp—pea protein, O—olive oil, P—papain).

Components (%)	Pp0-O0-P0	Pp0-O5-P0	Pp0-O10-P0	Pp1-O0-P0	Pp1-O5-P0	Pp1-O10-P0	Pp0-O0-P0.2	Pp0-O5-P0.2	Pp0-O10-P0.2	Pp1-O0-P0.2	Pp1-O5-P0.2	Pp1-O10-P0.2
Meat	86	81	76	85	80	75	85.8	80.8	75.8	84.8	79.8	74.8
TG	3	3	3	3	3	3	3	3	3	3	3	3
Salt	1	1	1	1	1	1	1	1	1	1	1	1
Water	10	10	10	10	10	10	10	10	10	10	10	10
**Pea Protein**	0	0	0	1	1	1	0	0	0	1	1	1
**Olive Oil**	0	5	10	0	5	10	0	5	10	0	5	10
**Papain**	0	0	0	0	0	0	0.2	0.2	0.2	0.2	0.2	0.2

## Data Availability

The raw data supporting the conclusions of this article will be made available by the authors on request.

## Supplementary Materials

The following supporting information can be downloaded from https://www.mdpi.com/article/10.3390/foods14142462/s1: Table S1. Multifactorial ANOVA results for firmness of ground and reconstituted meat samples (F-ratios and *p*-values); Table S2. Multifactorial ANOVA results (F-ratios and *p*-values) for the four texture parameters of the TM meat samples, TS samples, and their combination; Table S3. Data matrix used for the PCA.
