# Supplementary material for: Development of Texture-Modified Meat and Thickened Soup Combination for Oral Dysphagia Patients with Uniform Firmness and Solid Appearance
Source: Foods. 2025 Jul 14;14(14):2462. doi: 10.3390/foods14142462 (PMC12294358; doi:10.3390/foods14142462)
Supplement: Supplementary file 1 [file foods-14-02462-s001.zip › foods-3708020-supplementary.pdf]

Table S1. Multifactorial ANOVA results for firmness of ground and reconstituted meat samples (F-ratios and p-values).

	Firmness	P-value	F-ratio
B	F1: Pea Protein (%)	0.894	0.02
A	F2: Olive Oil (%)	**0.0001**	23.03
C	F3: Papain (%)	**0**	2002.49
AB		0.1689	2.07
AC		**0.0002**	18.53
BC		0.7332	0.12
ABC		0.1057	2.73

Table S2. Multifactorial ANOVA results (F-ratios and p-values) for the four texture parameters of TM meat samples, TS samples, and their combination.

		F1: Type of sample	F2: Force Group	AB
**Firmness**	P-value	**0.0005**	**0**	0.7065
	F-ratio	15.41	31.57	0.63
**Cohesiveness**	P-value	**0.0001**	**0.0002**	0.935
	F-ratio	24.02	16.41	0.28
**Consistency**	P-value	**0**	**0**	0.0885
	F-ratio	26.78	36.16	2.44
**Viscosity index**	P-value	**0**	**0.0001**	0.0535
	F-ratio	108.37	20.33	2.93

Table S3. Data matrix used for the PCA analysis.

Olive Oil (%)	Pea Protein (%)	Papain (%)	Firmness	Cohesiveness	Consistency	Index of Viscosity
0	0	0	85.287	-6.088	329.438	-9.003
5	0	0	61.978	-6.195	240.78	-8.316
10	0	0	61.706	-3.372	254.974	-4.383
0	1	0	72.593	-6.181	276.755	-7.314
5	1	0	65.431	-6.163	277.544	-8.629
10	1	0	65.247	-5.921	274.757	-7.234
0	0	0	81.75	-6.793	354.954	-12.372
5	0	0	54.204	-6.125	234.691	-9.277

10	0	0	53.901	-3.432	223.341	-3.689
0	1	0	76.095	-8.412	331.648	-9.63
5	1	0	54.55	-5.693	237.972	-10.074
10	1	0	60.809	-5.353	255.548	-7.183
0	0	0.2	3.803	-1.513	18.578	-2.922
5	0	0.2	2.833	-1.095	15.425	-2.135
10	0	0.2	3.04	-1.017	14.462	-2.239
0	1	0.2	4.405	-1.337	23.021	-2.952
5	1	0.2	2.95	-1.083	14.912	-2.338
10	1	0.2	2.775	-1.022	14.571	-1.956
0	0	0.2	2.846	-0.904	15.254	-2.174
5	0	0.2	2.637	-0.857	14.031	-2.047
10	0	0.2	2.422	-0.882	13.17	-2.053
0	1	0.2	4.063	-1.998	21.188	-2.578
5	1	0.2	2.537	-0.728	14.202	-1.566
10	1	0.2	2.65	-0.879	13.018	-1.964
